# *catena*-Poly[[[tetra­quazinc(II)]-μ-2,5-dihydroxy­benzene-1,4-diacetato-κ^2^ *O* ^1^:*O* ^4^] dihydrate]

**DOI:** 10.1107/S1600536808035514

**Published:** 2008-11-08

**Authors:** Liang Wang, Hongwei Zhang, Lin Yue, Zhaohui Zhang

**Affiliations:** a Department of Environmental Engineering, Polytechnic Tianjin University, Tianjin 300160, People’s Republic of China; b Tianjin Tianle International Engineering Consulting Co, Tianjin 300203, People’s Republic of China

## Abstract

The title compound, {[Zn(C_10_H_8_O_6_)(H_2_O)_4_]·2H_2_O}_*n*_, is a one-dimensional coordination polymer with 2,5-dihydroxy­benzene-1,4-diacetate acting as bridging ligand. The zigzag chains, extending parallel to [011], are further packed into a three-dimensional network by hydrogen bonds.

## Related literature

For related structures, see Ren *et al.* (2008 ▶); Cano *et al.* (1997 ▶); Sun *et al.* (2001 ▶); Zhao *et al.* (2004 ▶). 
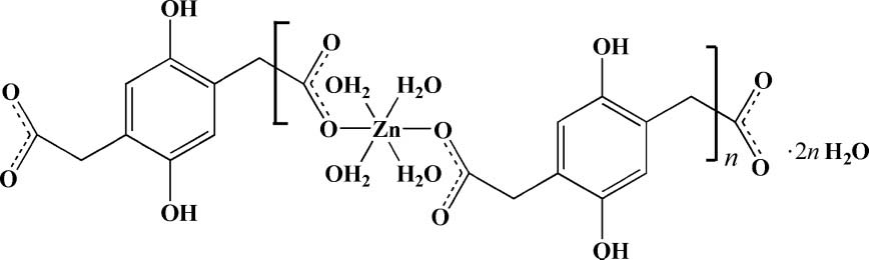


## Experimental

### 

#### Crystal data

[Zn(C_10_H_8_O_6_)(H_2_O)_4_]·2H_2_O
*M* *_r_* = 397.63
Monoclinic, 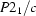

*a* = 11.122 (2) Å
*b* = 7.5176 (15) Å
*c* = 8.6417 (17) Å
β = 95.12 (3)°
*V* = 719.7 (2) Å^3^
*Z* = 2
Mo *K*α radiation
μ = 1.77 mm^−1^
*T* = 113 (2) K
0.32 × 0.24 × 0.10 mm

#### Data collection

Rigaku Saturn diffractometer
Absorption correction: multi-scan (*CrystalStructure*; Rigaku/MSC, 2005 ▶) *T*_min_ = 0.601, *T*_max_ = 0.843
6863 measured reflections
1833 independent reflections
1405 reflections with *I* > 2σ(*I*)
*R*_int_ = 0.041

#### Refinement

*R*[*F* ^2^ > 2σ(*F* ^2^)] = 0.032
*wR*(*F* ^2^) = 0.117
*S* = 1.17
1833 reflections
125 parameters
9 restraints
H atoms treated by a mixture of independent and constrained refinement
Δρ_max_ = 0.60 e Å^−3^
Δρ_min_ = −0.66 e Å^−3^

### 

Data collection: *CrystalStructure* (Rigaku/MSC, 2005 ▶); cell refinement: *CrystalStructure*; data reduction: *CrystalStructure*; program(s) used to solve structure: *SHELXS97* (Sheldrick, 2008 ▶); program(s) used to refine structure: *SHELXL97* (Sheldrick, 2008 ▶); molecular graphics: *ORTEP-3* (Farrugia, 1997 ▶); software used to prepare material for publication: *CrystalStructure*.

## Supplementary Material

Crystal structure: contains datablocks global, I. DOI: 10.1107/S1600536808035514/bq2094sup1.cif

Structure factors: contains datablocks I. DOI: 10.1107/S1600536808035514/bq2094Isup2.hkl

Additional supplementary materials:  crystallographic information; 3D view; checkCIF report

## Figures and Tables

**Table 1:** Hydrogen-bond geometry (Å, °)

*D*—H⋯*A*	*D*—H	H⋯*A*	*D*⋯*A*	*D*—H⋯*A*
O3—H3⋯O6^i^	0.84	1.92	2.725 (3)	160
O4—H4*A*⋯O2^ii^	0.857 (10)	1.823 (15)	2.616 (3)	153 (3)
O4—H4*A*⋯O1^ii^	0.857 (10)	2.45 (3)	3.011 (3)	123 (3)
O4—H4*B*⋯O6^iii^	0.859 (10)	1.925 (10)	2.783 (3)	176 (3)
O5—H5*A*⋯O3^iv^	0.853 (10)	2.000 (15)	2.828 (3)	164 (3)
O5—H5*B*⋯O4^iii^	0.855 (10)	1.956 (15)	2.787 (3)	164 (3)
O6—H6*A*⋯O2^v^	0.836 (10)	2.11 (3)	2.781 (3)	137 (3)
O6—H6*B*⋯O1	0.843 (10)	1.908 (15)	2.721 (3)	162 (3)

Symmetry codes: (i) 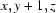; (ii) 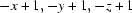; (iii) 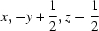; (iv) 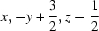; (v) 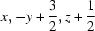.

**Fig. 1.**
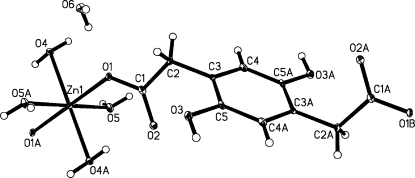
The molecular structure of the title compound with 30% probability displacement ellipsoids. Symmetry code: A - x, -y + 2 - z.

**Fig. 2.**
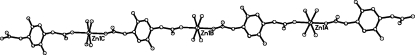
One-dimensional chain structure of the title compound. H atoms and lattice water molecules are omitted for clarity. Symmetry code: (A) x, y, z; (B) - x, -y + 2 - z; (C) 1 - x, 1 - y, -z.

**Fig. 3.**
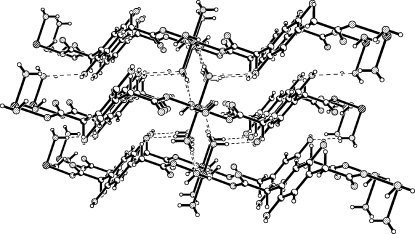
The packing diagram of the title compound.

## supplementary crystallographic information

### Comment

Rigid carboxylato ligands, such as benzene-carboxylic acid, pyridine-carboxylic acid, *etc*. have been widely utilized to synthesize coordination polymers because they can link metal ions *via* one carboxyl group or *via* the aroma rings, leading plentiful varieties of structures (Cano *et al.*, 1997; Sun *et al.*, 2001; Zhao, *et al.*, 2004). In contrast, flexible aroma-carboxylic acid and their complexes are less studied comparing to the rigid ones. (Ren *et al.*, 2008)

In this contribution, a flexible ligand, 2,5-dihydroxy-*p*-benzenediacetic acid (H_2_dba), was selected to construct coordination polymer, and the title complex was obtained under solvothermal conditions.

The Zn(II) ion in the title compound is coordinated by two oxygen atoms from dba anions in the apical sites and four water molecules in the equatorial plane (Fig. 1). The Zn(II) ions are linked through dba dianion forming one-dimensional chain (Fig. 2). Furthermore, the chains are packed into three-dimensional supermolecular moiety by O—H···O H-bonds (Fig. 3).

### Experimental

A mixture of Zn(Ac)_2_2H_2_O (0.5 mmol, 109.8 mg), H_2_dba (0.5 mmol, 133.0 mg), 10 ml THF and 10 ml water was put into a 25 ml acid digestion bomb and heated at 80°C for three days. After cooling to room temperature, the title compound (56% yield based on Zn(II) salt) was obtained. Elemental analysis (%) for the title compound C_10_H_20_ZnO_12_: found: C, 29.94; H, 4.96; N, 0. Calc.: C, 30.20; H, 5.07; N, 0.

### Refinement

The carboxyl H and aromatic H were placed in calculated positions and refined in riding mode with *U*_iso_(H) = 1.2*U*_eq_(C). Water H atoms were located in a difference Fourier map and refined as riding in as-found relative positions with *U*_iso_(H) = 1.5*U*_eq_(O).

### Crystal data

**Table:**

[Zn(C_10_H_8_O_6_)(H_2_O)_4_]·2H_2_O	*F*_000_ = 412
*M**_r_* = 397.63	*D*_x_ = 1.835 Mg m^−^^3^
Monoclinic, *P*2_1_/*c*	Mo *K*α radiation λ = 0.71073 Å
Hall symbol: -P2ybc	Cell parameters from 1803 reflections
*a* = 11.122 (2) Å	θ = 2.9–28.6º
*b* = 7.5176 (15) Å	µ = 1.77 mm^−^^1^
*c* = 8.6417 (17) Å	*T* = 113 (2) K
β = 95.12 (3)º	Prism, colorless
*V* = 719.7 (2) Å^3^	0.32 × 0.24 × 0.10 mm
*Z* = 2	

### Data collection

**Table:**

Rigaku Saturn diffractometer	1833 independent reflections
Radiation source: rotating anode	1405 reflections with *I* > 2σ(*I*)
Monochromator: graphite	*R*_int_ = 0.041
*T* = 113(2) K	θ_max_ = 28.6º
ω scans	θ_min_ = 1.8º
Absorption correction: multi-scan(CrystalStructure; Rigaku/MSC, 2005)	*h* = −14→14
*T*_min_ = 0.601, *T*_max_ = 0.843	*k* = −10→10
6863 measured reflections	*l* = −10→11

### Refinement

**Table:**

Refinement on *F*^2^	9 restraints
Least-squares matrix: full	H atoms treated by a mixture of independent and constrained refinement
*R*[*F*^2^ > 2σ(*F*^2^)] = 0.032	*w* = 1/[σ^2^(*F*_o_^2^) + (0.0565*P*)^2^ + 0.7652*P*] where *P* = (*F*_o_^2^ + 2*F*_c_^2^)/3
*wR*(*F*^2^) = 0.117	(Δ/σ)_max_ < 0.001
*S* = 1.17	Δρ_max_ = 0.60 e Å^−^^3^
1833 reflections	Δρ_min_ = −0.66 e Å^−^^3^
125 parameters	Extinction correction: none

### Special details

**Table:**

Geometry. All e.s.d.'s (except the e.s.d. in the dihedral angle between two l.s. planes) are estimated using the full covariance matrix. The cell e.s.d.'s are taken into account individually in the estimation of e.s.d.'s in distances, angles and torsion angles; correlations between e.s.d.'s in cell parameters are only used when they are defined by crystal symmetry. An approximate (isotropic) treatment of cell e.s.d.'s is used for estimating e.s.d.'s involving l.s. planes.
Refinement. Refinement of *F*^2^ against ALL reflections. The weighted *R*-factor *wR* and goodness of fit *S* are based on *F*^2^, conventional *R*-factors *R* are based on *F*, with *F* set to zero for negative *F*^2^. The threshold expression of *F*^2^ > σ(*F*^2^) is used only for calculating *R*-factors(gt) *etc*. and is not relevant to the choice of reflections for refinement. *R*-factors based on *F*^2^ are statistically about twice as large as those based on *F*, and *R*- factors based on ALL data will be even larger.

### Fractional atomic coordinates and isotropic or equivalent isotropic displacement parameters (Å^2^)

**Table:**

	*x*	*y*	*z*	*U*_iso_*/*U*_eq_	
Zn1	0.5000	0.5000	0.5000	0.00908 (16)	
O1	0.33235 (19)	0.5544 (3)	0.5773 (2)	0.0106 (4)	
O2	0.30469 (19)	0.8254 (3)	0.4764 (3)	0.0142 (5)	
O3	0.17185 (19)	1.0259 (3)	0.7503 (3)	0.0124 (5)	
H3	0.1832	1.1332	0.7746	0.019*	
O4	0.4629 (2)	0.2263 (3)	0.5260 (3)	0.0121 (4)	
H4A	0.5325 (13)	0.207 (5)	0.494 (4)	0.018*	
H4B	0.4037 (18)	0.197 (5)	0.460 (3)	0.018*	
O5	0.4263 (2)	0.5039 (3)	0.2703 (3)	0.0138 (4)	
H5A	0.3500 (11)	0.517 (4)	0.268 (4)	0.021*	
H5B	0.449 (3)	0.426 (4)	0.207 (4)	0.021*	
C1	0.2689 (3)	0.6913 (4)	0.5470 (3)	0.0103 (6)	
C2	0.1445 (3)	0.6927 (4)	0.6029 (4)	0.0107 (6)	
H2A	0.1014	0.5838	0.5647	0.013*	
H2B	0.1525	0.6879	0.7178	0.013*	
C3	0.0690 (2)	0.8511 (4)	0.5524 (3)	0.0090 (6)	
C4	−0.0186 (3)	0.8388 (4)	0.4269 (3)	0.0103 (6)	
H4	−0.0321	0.7277	0.3758	0.012*	
C5	0.0863 (2)	1.0146 (4)	0.6249 (3)	0.0095 (5)	
O6	0.2650 (2)	0.3550 (3)	0.8178 (3)	0.0133 (4)	
H6A	0.246 (3)	0.426 (4)	0.886 (3)	0.020*	
H6B	0.286 (3)	0.395 (4)	0.733 (2)	0.020*	

### Atomic displacement parameters (Å^2^)

**Table:**

	*U*^11^	*U*^22^	*U*^33^	*U*^12^	*U*^13^	*U*^23^
Zn1	0.0073 (2)	0.0100 (3)	0.0101 (3)	0.00122 (18)	0.00169 (16)	0.00064 (19)
O1	0.0082 (10)	0.0111 (10)	0.0125 (10)	0.0037 (8)	0.0015 (8)	0.0024 (8)
O2	0.0097 (10)	0.0127 (10)	0.0204 (12)	0.0011 (8)	0.0035 (9)	0.0034 (9)
O3	0.0108 (10)	0.0131 (11)	0.0125 (11)	−0.0002 (8)	−0.0035 (8)	−0.0011 (8)
O4	0.0086 (9)	0.0104 (10)	0.0177 (11)	0.0004 (8)	0.0032 (8)	0.0010 (8)
O5	0.0111 (10)	0.0182 (11)	0.0117 (11)	0.0018 (9)	−0.0007 (8)	−0.0026 (9)
C1	0.0089 (13)	0.0116 (14)	0.0104 (14)	0.0005 (11)	−0.0001 (11)	−0.0042 (11)
C2	0.0080 (13)	0.0126 (14)	0.0117 (14)	0.0043 (10)	0.0023 (11)	0.0046 (11)
C3	0.0049 (12)	0.0095 (13)	0.0132 (14)	0.0020 (10)	0.0043 (10)	0.0031 (11)
C4	0.0079 (12)	0.0156 (14)	0.0080 (14)	0.0006 (11)	0.0039 (10)	−0.0018 (11)
C5	0.0048 (11)	0.0159 (14)	0.0082 (13)	0.0023 (11)	0.0027 (9)	0.0011 (11)
O6	0.0159 (11)	0.0119 (11)	0.0123 (11)	−0.0003 (8)	0.0028 (9)	0.0001 (8)

### Geometric parameters (Å, °)

**Table:**

Zn1—O1	2.077 (2)	O5—H5B	0.855 (10)
Zn1—O1^i^	2.077 (2)	C1—C2	1.506 (4)
Zn1—O5^i^	2.080 (2)	C2—C3	1.500 (4)
Zn1—O5	2.080 (2)	C2—H2A	0.9900
Zn1—O4	2.115 (2)	C2—H2B	0.9900
Zn1—O4^i^	2.115 (2)	C3—C5	1.384 (4)
O1—C1	1.262 (3)	C3—C4	1.395 (4)
O2—C1	1.261 (4)	C4—C5^ii^	1.387 (4)
O3—C5	1.379 (3)	C4—H4	0.9500
O3—H3	0.8400	C5—C4^ii^	1.387 (4)
O4—H4A	0.857 (10)	O6—H6A	0.836 (10)
O4—H4B	0.859 (10)	O6—H6B	0.843 (10)
O5—H5A	0.853 (10)		
			
?···?	?		
			
O1—Zn1—O1^i^	180.0	Zn1—O5—H5B	119 (2)
O1—Zn1—O5^i^	89.12 (9)	H5A—O5—H5B	114.9 (18)
O1^i^—Zn1—O5^i^	90.88 (9)	O2—C1—O1	123.9 (3)
O1—Zn1—O5	90.88 (9)	O2—C1—C2	119.2 (3)
O1^i^—Zn1—O5	89.12 (9)	O1—C1—C2	116.8 (3)
O5^i^—Zn1—O5	180.0	C3—C2—C1	114.8 (2)
O1—Zn1—O4	88.16 (8)	C3—C2—H2A	108.6
O1^i^—Zn1—O4	91.84 (8)	C1—C2—H2A	108.6
O5^i^—Zn1—O4	87.08 (8)	C3—C2—H2B	108.6
O5—Zn1—O4	92.92 (9)	C1—C2—H2B	108.6
O1—Zn1—O4^i^	91.84 (8)	H2A—C2—H2B	107.6
O1^i^—Zn1—O4^i^	88.16 (8)	C5—C3—C4	118.1 (3)
O5^i^—Zn1—O4^i^	92.92 (9)	C5—C3—C2	121.4 (3)
O5—Zn1—O4^i^	87.08 (8)	C4—C3—C2	120.5 (3)
O4—Zn1—O4^i^	180.0	C5^ii^—C4—C3	121.3 (3)
C1—O1—Zn1	126.52 (19)	C5^ii^—C4—H4	119.4
C5—O3—H3	109.5	C3—C4—H4	119.4
Zn1—O4—H4A	86 (3)	O3—C5—C3	117.9 (3)
Zn1—O4—H4B	109 (3)	O3—C5—C4^ii^	121.4 (3)
H4A—O4—H4B	113.9 (17)	C3—C5—C4^ii^	120.7 (3)
Zn1—O5—H5A	109 (3)	H6A—O6—H6B	119.8 (19)
			
O5^i^—Zn1—O1—C1	118.5 (2)	C1—C2—C3—C5	−77.9 (4)
O5—Zn1—O1—C1	−61.5 (2)	C1—C2—C3—C4	100.0 (3)
O4—Zn1—O1—C1	−154.4 (2)	C5—C3—C4—C5^ii^	0.2 (5)
O4^i^—Zn1—O1—C1	25.6 (2)	C2—C3—C4—C5^ii^	−177.7 (3)
Zn1—O1—C1—O2	−7.4 (4)	C4—C3—C5—O3	179.1 (3)
Zn1—O1—C1—C2	173.89 (19)	C2—C3—C5—O3	−2.9 (4)
O2—C1—C2—C3	6.2 (4)	C4—C3—C5—C4^ii^	−0.2 (5)
O1—C1—C2—C3	−175.0 (3)	C2—C3—C5—C4^ii^	177.7 (3)

Symmetry codes: (i) −*x*+1, −*y*+1, −*z*+1; (ii) −*x*, −*y*+2, −*z*+1.

### Hydrogen-bond geometry (Å, °)

**Table:**

*D*—H···*A*	*D*—H	H···*A*	*D*···*A*	*D*—H···*A*
O3—H3···O6^iii^	0.84	1.92	2.725 (3)	160
O4—H4A···O2^i^	0.857 (10)	1.823 (15)	2.616 (3)	153 (3)
O4—H4A···O1^i^	0.857 (10)	2.45 (3)	3.011 (3)	123 (3)
O4—H4B···O6^iv^	0.859 (10)	1.925 (10)	2.783 (3)	176 (3)
O5—H5A···O3^v^	0.853 (10)	2.000 (15)	2.828 (3)	164 (3)
O5—H5B···O4^iv^	0.855 (10)	1.956 (15)	2.787 (3)	164 (3)
O6—H6A···O2^vi^	0.836 (10)	2.11 (3)	2.781 (3)	137 (3)
O6—H6B···O1	0.843 (10)	1.908 (15)	2.721 (3)	162 (3)

Symmetry codes: (iii) *x*, *y*+1, *z*; (i) −*x*+1, −*y*+1, −*z*+1; (iv) *x*, −*y*+1/2, *z*−1/2; (v) *x*, −*y*+3/2, *z*−1/2; (vi) *x*, −*y*+3/2, *z*+1/2.
